# Evaluation of Analgesic Effect of Caudal Epidural Tramadol, Tramadol-Lidocaine, and Lidocaine in Water Buffalo Calves (*Bubalus bubalis*)

**DOI:** 10.1155/2015/575101

**Published:** 2015-12-03

**Authors:** Ayman Atiba, Alaa Ghazy, Naglaa Gomaa, Tarek Kamal, Mustafa Shukry

**Affiliations:** ^1^Department of Surgery, Anesthesiology and Radiology, Faculty of Veterinary Medicine, Kafrelsheikh University, Kafrelsheikh 33516, Egypt; ^2^Department of Animal Medicine, Faculty of Veterinary Medicine, Kafrelsheikh University, Kafrelsheikh 33516, Egypt; ^3^Department of Biochemistry, Faculty of Veterinary Medicine, Kafrelsheikh University, Kafrelsheikh 33516, Egypt; ^4^Department of Physiology, Faculty of Veterinary Medicine, Kafrelsheikh University, Kafrelsheikh 33516, Egypt

## Abstract

Aim of this study was to compare the analgesic effect of tramadol and a combination of tramadol-lidocaine with that produced by lidocaine administration in the epidural space in buffalo calves. In a prospective randomized crossover study, ten male buffalo calves were used to compare the epidural analgesic effect of tramadol (1 mg/kg) and tramadol-lidocaine combination (0.5 mg/kg and 0.11 mg/kg, resp.) with that produced by 2% lidocaine (0.22 mg/kg). Loss of sensation was examined by pin-prick test. Onset time, duration, and degree of analgesia and ataxia were recorded after each treatment. Heart rate (HR), respiratory rate (RR), rectal temperature, and haematobiochemical parameters were recorded after all treatments. Time to onset and duration of analgesia, respectively, were as follows: tramadol 11 ± 2 min and 208 ± 15 min; tramadol-lidocaine 6 ± 2 min and 168 ± 9 min; lidocaine 4 ± 1 min and 67 ± 13 min. Onset time and duration were significantly longer with tramadol than the other treatments. Duration was significantly longer with tramadol-lidocaine than lidocaine. Ataxia was mildly observed in tramadol-lidocaine and was moderate in lidocaine. HR, RR, and rectal temperature did not differ significantly from baseline after any treatment. Haematobiochemical parameters returned to basal levels by 24 h after all treatments. This combination might be clinically useful to provide analgesia in buffalo for long-duration surgical procedures.

## 1. Introduction

Caudal epidural analgesia is frequently used in large animals for surgical procedures in the perineal region [1]. Lidocaine is the most commonly used local anesthetic drug in epidural analgesia but has a relatively short duration of action and may require readministration of the drug to allow completion of the procedure [2]. Epidural administration of a drug or a combination results in a much longer duration of action which will be more appropriate for procedures requiring long-duration analgesia [3].

Recently, tramadol as an adjuvant to local anesthetics has been found to improve the quality and extend the duration of analgesia in humans [4, 5]. Tramadol is a synthetic analogue of codeine that has been demonstrated to provide prolonged epidural analgesia in human [6]. Moreover, epidural tramadol has been used alone or in combination with lidocaine to produce longer analgesia in many animal species, including horses, cattle, goats, and donkeys [7–10]. To date, epidural injection of tramadol has not yet been reported in buffalo. Therefore, the purpose of this study was to determine and compare the time of onset and the duration of analgesia produced by tramadol and tramadol-lidocaine combination with that produced by lidocaine administration in the epidural space of buffalo calves.

## 2. Materials and Methods

In this study, the experimental protocol was approved by the Animal Care Committee of Kafrelsheikh University.

### 2.1. Animals

Ten male water buffalo calves (*Bubalus bubalis*) aged 7–10 months, weight 78–112 kg, were selected. All calves were deemed healthy by physical examination. The animals were fasted for 24 hours, and water was withheld for 6 hours prior to the start of the experiment.

The animals were restrained in standing position in the stanchion. The sacrococcygeal (S5-Co1) area of each calf was shaved and prepared aseptically. Epidural injection was made using a 20-gauge, 4-cm long hypodermic needle. Confirmation of proper epidural injection was based on the standard technique of loss of resistance to injection of 2 mL of air and the hanging drop technique. In crossover study, calves were randomly assigned to receive each of the three treatment protocols, with a period of at least 10-day interval between them in a Latin square design. Treatment 1 was tramadol (1 mg/kg) (Amadol, 100 mg/1 mL, injectable sterilized solution; Adwia Co., 10th of Ramadan City, Egypt). Treatment 2 was a combination of tramadol (0.5 mg/kg) and 2% lidocaine hydrochloride without epinephrine (0.11 mg/kg) (Debocaine 2% Al-Debeiky Pharmaceutical Industries Co., Egypt). Treatment 3 was 2% lidocaine (0.22 mg/kg). The volume of the drug was expanded to 4.0 mL by adding 0.9% normal saline solution. All drugs were injected slowly over a period of approximately 40 seconds.

**Figure 1:**
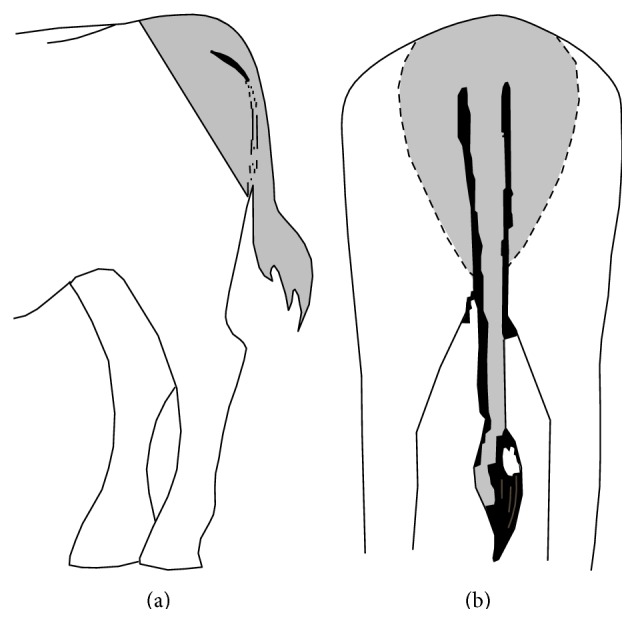
Schematic diagram from the left lateral view (a) and caudal view (b) of a buffalo calf showing the area was tested by a pin-prick test (applied at the perineal region, tail base, and upper hind limb area).

Time to the onset, duration, anatomical distribution, and degree of the analgesia were recorded. Analgesia was assessed by a pin-prick test (using a 21-gauge needle inserted through the skin into the underlying tissues) applied at the perineal region, tail base, and upper hind limb area (Figure 1). Time to the onset of analgesia was defined as a time interval (in minute) from the epidural injection of the drug to loss of response to pin-prick in the perineal region. The duration time of analgesia was defined as a time interval (in minute) from loss and reappearance of pain response to pin-prick in the perineal region. In the stanchions, the response was measured each minute until no reaction occurred, and then at 10-minute intervals until a response was observed. The degree of analgesia was graded subjectively by observing the response of the animal to the pin-prick test on a scale from 0 to 3 according to the following scoring system: 0 = no analgesia (strong avoidance response to pin-prick); 1 = mild analgesia (weak avoidance response to pin-prick); 2 = moderate analgesia (occasional mild avoidance response to pin-prick); 3 = complete analgesia (no avoidance response to pin-prick). To avoid any bias or potential manipulation of data, the same investigator assessed the analgesia in all calves and was blind to the treatment given.

The presence of ataxia was examined by walking animals out of the stanchions at 10-minute intervals until the end of the study. The ataxia was graded on a 0 to 3 scale according to Grubb et al. 2002 [11], using the following scoring system: 0 = normal (walking without staggering); 1 = mild (slight stumbling, easily able to continue walking); 2 = moderate (marked stumbling, walking but very ataxic); 3 = severe (animal unable to stand and falling). Person, unaware of the drugs used, made the observations.

Heart rate (HR) was measured by counting the heart beats over the cardiac area using a stethoscope. Respiratory rate (RR) was measured by counting chest movements per minute and rectal temperature (°C) was measured with a digital thermometer. HR, RR, and rectal temperature were recorded before (baseline, 0) and at 10-minute intervals for 2 hours after the administration of each treatment protocol. Blood samples were collected from the jugular vein for haematobiochemical parameters before (0 minute), at 30, 60, 90, 120, minutes, and 24-hour intervals after administration of drugs. For haematology, 3 mL venous blood was collected in test tubes containing EDTA. Hematological parameters including haemoglobin (Hb), packed cell volume (PCV%), total erythrocytes count (RBCs), and total leukocyte count (TLC) were examined. For biochemical parameters, 7 mL venous blood was collected in test tubes and serum was separated. Biochemical parameters including blood glucose, blood urea nitrogen (BUN) alanine aminotransferase (ALT), and alkaline phosphatase (ALP) were examined.

### 2.2. Statistical Analyses

All data were presented as mean ± SD. The data for time of onset and duration of analgesia, HR, RR, rectal temperature, and haematobiochemical parameters were analyzed by ANOVA (analysis of variance) and Duncan's test as a post hoc. Statistical analysis was undertaken using Graphpad Prism version 5 software program. A value of *p* < 0.05 was considered significant.

## 3. Results

Onset time of analgesia was significantly (*p* < 0.05) longer after tramadol than after the tramadol-lidocaine combination or lidocaine alone (Table 1). The duration of analgesia was significantly longer in animals that received tramadol (*p* = 0.038) or the combination of tramadol-lidocaine (*p* = 0.043) as compared with animals that received lidocaine alone and the duration of action of the tramadol was significantly greater than for tramadol-lidocaine combination (*p* = 0.044) (Table 1).

**Figure 2:**
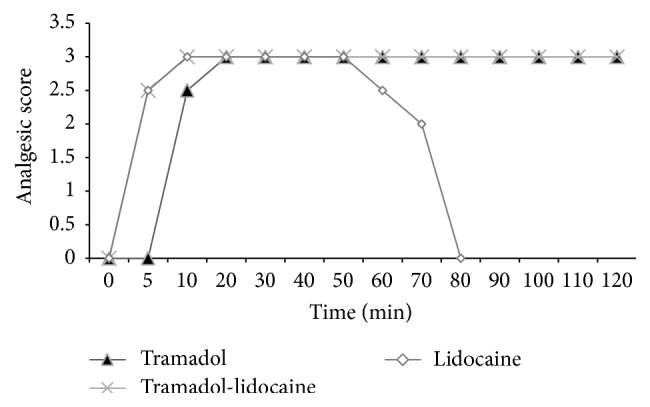
Degree of analgesia at the perineal region and tail base after epidural analgesia of tramadol, tramadol-lidocaine combination, and lidocaine in buffalo calves.

The results of this study demonstrated that all of the three treatments were effective in producing analgesia in the perineal region, tail base, and upper hind limb regions in all experimental animals but at different times. Analgesia at the perineal region and tail base (Figure 2) increased gradually and complete analgesia (score 3) was recorded from 10 minutes in lidocaine and tramadol-lidocaine groups while recorded from 20 minutes onwards in tramadol group. Analgesia started to wean off gradually and was lost by 80 minutes in lidocaine group. Analgesia of tramadol and tramadol-lidocaine groups showed complete analgesia until the end of the observation time. Analgesia at upper hind limb region was ranged from mild to moderate (score 1 to 2) and was observed from 10 to 60 minutes in lidocaine group. In animal of tramadol-lidocaine and tramadol groups moderated analgesia (score 2) was observed 20 and 30 minutes onwards, respectively, until the end of the observation time.

Ataxia was observed in all calves following epidural administration of lidocaine and a tramadol-lidocaine combination. All animals in the lidocaine group showed moderate ataxia (score 2) which started after 15 minutes and lasted for 35 minutes, while animals in tramadol-lidocaine combination group showed mild ataxia (score 1) which started after 20 minutes and lasted for 60 minutes from the time of injection. Animals of the tramadol group showed no signs of ataxia and animals of all groups remained in standing position all over the time of observation.

**Figure 3:**
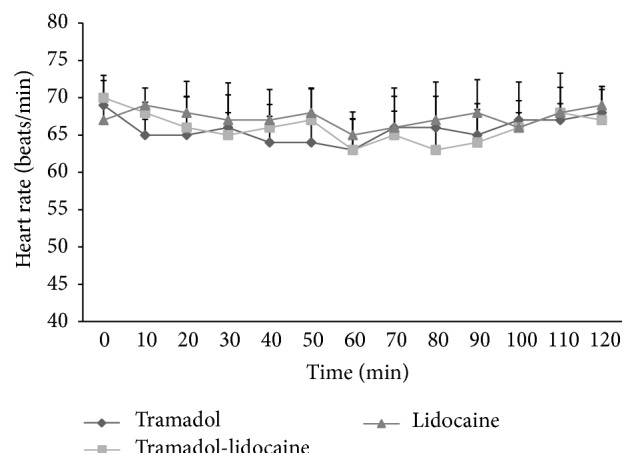
Mean heart rate (beats/minute) after epidural analgesia of tramadol, tramadol-lidocaine combination, and lidocaine in buffalo calves (mean ± SD in 10 calves). Error bars indicate the standard deviation of the mean.

**Figure 4:**
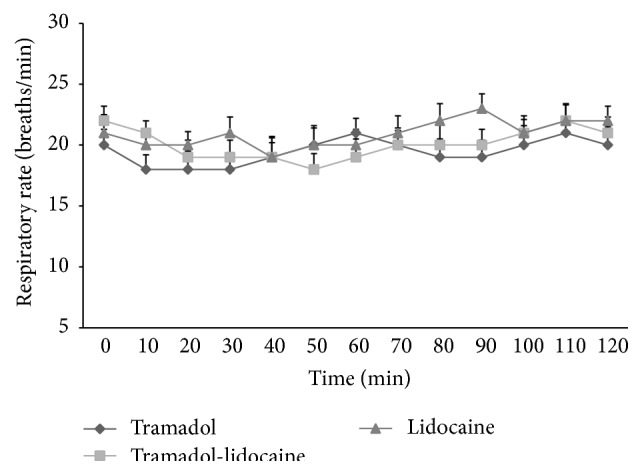
Mean respiratory rate (breaths/minute) after epidural analgesia of tramadol, tramadol-lidocaine combination, and lidocaine in buffalo calves (mean ± SD in 10 calves). Error bars indicate the standard deviation of the mean.

The HR (Figure 3), RR (Figure 4), and rectal temperatures did not differ significantly (*p* > 0.05) from baseline values following any treatment. Hematological parameters: a significant decrease in Hb, PCV%, RBCs, and TLC was recorded after 30 minutes, which persisted up to 120-minute intervals as compared to base value in all groups. However, values returned to basal level by 24 hours (Table 2). Biochemical parameters: a significant (*p* < 0.01) increase in glucose concentration from 30- to 120-minute intervals was recorded. A significant increase in BUN levels was observed in all animals after different treatments. ALP level concentration showed no significant difference among different groups, while ALT level concentration showed a significant increase at different time points of treatment. However, biochemical parameters returned to basal levels after 24 hours of treatments (Table 2).

## 4. Discussion

There are currently only few published studies about epidural analgesia in buffalo [12–15]. Epidural use of tramadol has been reported in human and different animal species to provide prolonged duration of analgesia without serious side effects [4, 7, 16]. The present study is the first to investigate the analgesic effect of tramadol and tramadol-lidocaine combination given by an epidural injection to buffalo calves. The dose of tramadol chosen was based on the previous studies, where tramadol was used epidurally in horses, cattle, goats, and donkeys at doses of 1 mg/kg [7–10].

In the current study, the onset time of analgesia was prolonged with tramadol alone, compared with tramadol-lidocaine combination and lidocaine alone. Similar findings have been reported previously in horses, cattle, goats, and donkeys [7–10]. Moreover, epidural tramadol alone or tramadol-lidocaine combination produced significantly longer duration of analgesia in buffalo calves compared with lidocaine alone. These results are in agreement with the previous reports that assessed the use of tramadol with the local anesthetic agents. They concluded that the duration of analgesia was significantly extended following the administration of epidural tramadol without any side effects [7–10].

Vasoconstrictors (mainly epinephrine) were used to increase the depth or duration of local anesthetics for epidural block [17]. However, all the previous studies compared the analgesic effect of epidural tramadol with plain lidocaine without epinephrine [8–10]. Similarly, the current study compared tramadol with plain lidocaine. Further studies are required to compare epidural tramadol with lidocaine-epinephrine.

Tramadol is an opioid analgesic with central effects like codeine and morphine [6]. Mechanism of action by which tramadol induced analgesia could be its action on opioid receptors and through inhibition of the reuptake of norepinephrine and serotonin [6, 16]. Tramadol alone produced longer duration of analgesia than tramadol-lidocaine combination. The vasodilatation due to sympathetic nerve block produced by epidurally injected lidocaine decreased the duration of analgesia [18]. In the present study, also shortened duration of analgesia in the tramadol-lidocaine combination compared with tramadol alone may be related to the possibility of vasodilation due to presence of lidocaine and/or the half dose of tramadol used in this combination [8, 10]. Further research is necessary to determine the effect of various doses of tramadol in epidural administration in buffalo calves.

Complete analgesia at the perineal region and tail base was recorded after all treatments plausibly due to higher concentrations of the drugs in the sacrococcygeal space (site of epidural injection of drugs). Mild to moderate degree of analgesia at upper hind limb was probably attributed to the decreased concentration of the drugs anteriorly. In this study, mild to moderate ataxia was observed in buffalo following epidural administration of lidocaine and tramadol-lidocaine combination, which is as expected due to the blocking effect of lidocaine on both the sensory and motor nerves [2], while no ataxia was observed after epidural tramadol administration. These results are similar to those previously reported in cattle and other species [7–9].

The HR, RR, and rectal temperature were not significantly different in comparison with baseline values throughout the study in all treatments. This is in agreement with previous results reported following extradural tramadol in combination with lidocaine in cattle [8].

The measurement of haematobiochemical parameters following epidural tramadol has not yet been estimated in the previous animal studies. In the present study, the decrease in hematological parameters including Hb, PCV%, RBCs, and TLC after epidural analgesia might be due to shifting of fluids from extravascular compartment to intravascular compartment to compensate normal cardiac output [19] and might be due to pooling of blood cells in the reservoir organs like spleen, secondary to decrease sympathetic activity [12]. The present finding is similar to previous findings after epidural xylazine combined with lignocaine in cow calves [20] and with our previous study after epidural lidocaine-neostigmine combination in buffalo calves [13]. A significant increase in glucose levels was observed from 30- to 120-minute time points after all treatments. But, at 24 hours, glucose levels returned to basal levels. The exact mechanism of hyperglycaemia induced after epidural tramadol, tramadol-lidocaine combination, and lidocaine was not investigated in the current study. Mirakhur et al. 1984 [14] suggested that hyperglycaemia may be due to a rise in adrenocortical hormones during stress. The increase in BUN levels in all animals after all treatments may be as a result of increased hepatic urea production from amino acid degradation as reported earlier [15]. Similar changes in BUN levels have also been reported after epidural lidocaine-neostigmine in buffalo calves [13]. A significant increase in ALP and ALT levels after all treatments might be related to some alterations in cell membrane permeability, which may permit these enzymes to leak from the cells with intact membranes [21]. As the values returned to baseline levels at 24 hours of observation, the possibility of pathological changes in the liver could therefore be ruled out. Similar findings were reported after xylazine, lignocaine, and their combination for lumbar epidural analgesia in water buffalo calves [15].

In conclusion, the results of this study suggested that the combination of tramadol-lidocaine produced good analgesia with relatively rapid onset compared with tramadol alone and a longer duration compared with lidocaine alone in the perineal area. This combination may be useful in clinical practice for longer duration of surgical and obstetrical procedures. However, further studies are required to determine the utility of this combination for surgical procedures before final recommendations can be made.

## Figures and Tables

**Table 1:** Anesthetic indices of epidurally administered tramadol (1.0 mg/kg), tramadol-lidocaine (0.5 and 0.11 mg/kg, resp.), and lidocaine (0.22 mg/kg) in buffalo calves (mean ± SD; *n* = 10).

Indices	Tramadol	Tramadol-lidocaine	Lidocaine
Onset of analgesia (min)	11 ± 2^a^	6 ± 2	4 ± 1
Duration of analgesia (min)	208 ± 15	168 ± 9^c^	67 ± 13^b^

^a^Significant differences between onset of tramadol alone with lidocaine and tramadol-lidocaine combination. ^b^Significant differences between duration of lidocaine alone with tramadol-lidocaine combination and tramadol alone. ^c^Significant differences between duration of tramadol-lidocaine combination and lidocaine alone.

**Table 2:** Hematobiochemical parameters after caudal epidural administration of tramadol alone (T), tramadol-lidocaine combination (TL), and lidocaine alone (L) in buffalo calves (mean ± SD, *n* = 10).

Parameter	Group	Time interval					
		0 min	30 min	60 min	90 min	120 min	24 h
Hb (gm/dL)	T	10.25 ± 1.3^a^	8.4 ± 1.2^b^	7.2 ± 1.3^c^	6.1 ± 0.3^cd^	6.2 ± 1.0^cd^	10.4 ± 0.3^a^
	TL	10.1 ± 1.8^a^	8.0 ± 0.7^b^	7.15 ± 1.2^c^	6.75 ± 1.0^c^	6.81 ± 0.7^c^	10.5 ± 0.3^a^
	L	9.7 ± 1.4^a^	7.24 ± 1.2^c^	7.20 ± 1.1^c^	6.82 ± 0.8^c^	6.05 ± 1.2^cd^	9.9 ± 0.5^a^

PCV%	T	26.8 ± 1.3^ab^	23.7 ± 2.1^c^	22.1 ± 1.3^c^	20.9 ± 0.3^d^	20.2 ± 1.1^d^	26.9 ± 1.2^ab^
	TL	28.2 ± 1.2^a^	24.0 ± 1.4^c^	22.02 ± 0.8^c^	20.4 ± 0.2^d^	20.0 ± 1.5^d^	28. 2 ± 0.2^a^
	L	28.8 ± 0.7^a^	23.24 ± 0.4^c^	21.9 ± 0.2^cd^	21.20 ± 0.8^d^	22.03 ± 0.6^c^	28.01 ± 1.0^a^

RBCs (10^6^/mm^3^)	T	6.4 ± 0.5^a^	5.5 ± 0.1^ab^	5.0 ± 0.3^b^	4.7 ± 0.1^b^	4.3 ± 0.1^b^	6.6 ± 0.2^a^
	TL	5.7 ± 0.3^ab^	4.9 ± 0.3^b^	4.4 ± 0.5^b^	4.1 ± 0.2^b^	3.75 ± 0.4^bc^	5.6 ± 0.5^ab^
	L	6.4 ± 0.3^a^	4.9 ± 0.5^b^	4.5 ± 0.2^b^	4.01 ± 0.2^b^	4.04 ± 0.5^b^	6.2 ± 0.2^a^

TLC (10^3^/mm)	T	8.79 ± 0.52^ab^	7.7 ± 0.02^b^	7.91 ± 0.23^b^	7.0 ± 0.3^bc^	6.9 ± 0.02^bc^	9.25 ± 0.52^a^
	TL	9.22 ± 0.31^a^	8.5 ± 0.2^b^	7.5 ± 0.6^bc^	6.2 ± 1.1^c^	6.0 ± 0.2^c^	9.5 ± 0.2^a^
	L	9.5 ± 0.6^a^	8.1 ± 0.4^b^	7.3 ± 0.3^bc^	6.1 ± 0.2^c^	6.0 ± 0.5^c^	9.3 ± 0.7^a^

Blood glucose (mg/dL)	T	70.2 ± 1.5^d^	75.1 ± 0.2^b^	76.2 ± 0.6^ab^	76.5 ± 0.3^ab^	79.1 ± 0.1^a^	69.6 ± 1.0^d^
	TL	69.5 ± 1.5^d^	73.1 ± 2.3^c^	78.2 ± 1.1^a^	79.2 ± 0.4^a^	79.5 ± 1.5^a^	69.7 ± 0.8^d^
	L	70.5 ± 2.0^d^	75.2 ± 2.5^b^	75.4 ± 2.1^b^	77.3 ± 0.2^ab^	78.7 ± 2.5^a^	69.7 ± 2.0^d^

BUN (mg/dL)	T	29.2 ± 2.2^a^	29.7 ± 1.2^a^	29.9 ± 0.3^a^	29.9 ± 0.2^a^	30.4 ± 1.0^a^	28.3 ± 2.5^a^
	TL	24.2 ± 2.3^c^	25.1 ± 2.1^c^	27.2 ± 0.1^ab^	29.3 ± 0.4^a^	29.5 ± 0.9^a^	23.5 ± 2.2^c^
	L	25.4 ± 1.2^c^	26.2 ± 1.2^b^	27.5 ± 1.2^ab^	29.3 ± 1.1^a^	29.5 ± 1.3^a^	25.4 ± 1.4^c^

ALP (Unit/mL)	T	84.2 ± 2.2^a^	84.1 ± 1.2^a^	85.1 ± 2.2^a^	85.0 ± 0.2^a^	85.3 ± 1.2^a^	84.1 ± 2.0^a^
	TL	83.3 ± 1.2^ab^	84.1 ± 2.3^a^	85.0 ± 2.1^a^	85.5 ± 1.2^a^	85.5 ± 2.2^a^	83.4 ± 2.0^ab^
	L	83.2 ± 1.4^ab^	84.5 ± 2.2^a^	85.4 ± 2.0^a^	85.3 ± 0.6^a^	85.1 ± 2.5^a^	82.2 ± 1.0^ab^

ALT (Unit/mL)	T	27.2 ± 0.2^bc^	28.9 ± 0.2^a^	28.91 ± 0.3^a^	28.95 ± 0.3^a^	29.01 ± 0.2^a^	26.2 ± 0.1^a^
	TL	24.3 ± 0.3^c^	27.5 ± 1.0^bc^	28.9 ± 0.5^a^	29.01 ± 0.2^a^	29.2 ± 0.5^a^	23.9 ± 0.4^c^
	L	25.4 ± 0.7^c^	26.5 ± 1.6^c^	28.1 ± 0.4^ab^	28.5 ± 0.3^a^	28.9 ± 0.3^a^	25.2 ± 0.5^c^

Values with different letters differ significantly (*p* < 0.05) among groups.
